# Application of Ultraviolet-Visible Absorption Spectroscopy with Machine Learning Techniques for the Classification of Cretan Wines

**DOI:** 10.3390/foods10010009

**Published:** 2020-12-22

**Authors:** Aggelos Philippidis, Emmanouil Poulakis, Renate Kontzedaki, Emmanouil Orfanakis, Aikaterini Symianaki, Aikaterini Zoumi, Michalis Velegrakis

**Affiliations:** 1 Institute of Electronic Structure and Laser, Foundation for Research and Technology-Hellas (IESL-FORTH), 700 13 Heraklion, Greece; filagg@iesl.forth.gr (A.P.); mpoulakis@iesl.forth.gr (E.P.); renate.kontz@windowslive.com (R.K.); manolis.orfanak@gmail.com (E.O.); ksimianaki@gmail.com (A.S.); azoumi@iesl.forth.gr (A.Z.); 2 Department of Chemistry, University of Crete, 700 13 Heraklion, Crete, Greece; 3 Department of Materials Science and Technology, University of Crete, 700 13 Heraklion, Greece

**Keywords:** wine discrimination, grape varieties, wine maturation, aging, container, oak barrel, acacia barrel, Greek wines, Cretan wines, machine learning, multivariate analysis, chemometrics, OPLS-DA, ultraviolet–visible spectroscopy, spectral analysis

## Abstract

The present study was aimed at the identification, differentiation and characterization of red and white Cretan wines, which are described with Protected Geographical Indication (PGI), using ultraviolet–visible absorption spectroscopy. Specifically, the grape variety, the wine aging process and the role of barrel/container type were investigated. The combination of spectroscopic results with machine learning-based modelling demonstrated the use of absorption spectroscopy as a facile and low-cost technique in wine analysis. In this study, a clear discrimination among grape varieties was revealed. Moreover, a grouping of samples according to aging period and container type of maturation was accomplished, for the first time.

## 1. Introduction

The study and characterization of wine has drawn particular interest due to the continuous growth of its commercial interest. Wine is a complicated mixture of different chemical compounds, among which polyphenols have a dominant role to its characteristics and quality. The main origin of phenols is the grapes; however, smaller amounts may also be extracted from the wood barrels used as wine containers. During the wine aging in barrels, the slow and continuous diffusion of oxygen through the wood pores leads to the extraction of chemical substances, mainly ellagitannins [1]. Although grape variety, climate, and soil are considered to be the most dominant factors affecting wine quality, different maturation procedures may also influence the final product. Aging in wooden barrels is considered to be a common practice for both red and white wines. The wine maturation in oak barrels is a widespread method for the production of high-quality wine. In the recent years, other types of wood, such as acacia, has been used for wine aging, contributing to different organoleptic characteristics of wine [2,3]. An alternative technique used in order to reduce the costs of the purchase of a wood barrel is the addition of oak chips in stainless steel containers [4,5,6]. This procedure also accelerates the wine aging in stainless steel containers. The use of different type of wooden barrels results in the release of different chemical compounds during wine maturation. This is due to the different chemical properties of woods [2]. The type of barrel in combination with the contact time alters the sensory characteristics of wines such as color, aromas, structure and flavor, which are related to the phenolic composition of the samples [7,8]. 

Several studies have been performed on the evolution of physicochemical and sensory characteristics of wines during barrel aging [8,9,10]. Various analytical techniques have been developed in order to study wine discrimination based on the grape variety used for winemaking, aging process, vintage and geographical origin. These analytical techniques and instrumentation are mainly based on chromatographic methods [11,12,13]. Other sophisticated methods, such as Inductively Coupled Plasma Mass Spectrometry (ICP-MS) and Nuclear Magnetic Resonance (NMR) spectroscopy have been used in wine analysis as well [14,15,16]. Even though these techniques provide valuable information and precise analytical data on the wine analysis, they are often expensive and time consuming. Thus, in recent years there has been a growing interest to develop fast, low-cost, non-destructive techniques, which demand no, or minimal sample pretreatment. 

Laser-induced breakdown spectroscopy in combination with classification methods have been used to discriminate grape seeds [17]. Fourier Transform Infrared spectroscopy (FT-IR) combined with chemometrics has been employed to discriminate wines. A good discrimination of the samples according to grape variety, the container type and the aging time was achieved [18]. Another technique, Ultraviolet–Visible (UV-Vis) absorption spectroscopy, fulfils the above criteria (fast, low cost, no sample pretreatment) and can be applied to the analysis of wines and the qualitative detection of phenolic composition. The absorption spectrum in the UV-Vis region may be used as a fingerprint in wine discrimination. Most of the studies employing UV-Vis spectroscopy are based on absorbance values in specific wavelengths in order to quantify families of phenolic compounds and color characteristics [19,20]. Combining the UV-Vis spectroscopic technique with chemometrics has been used for the discrimination of wines of different varietal, geographical origin and aging process [21,22,23]. Fluorescence technique has also been used for wine identification. Front-face fluorescence spectroscopy in combination with PARAllel FACtor Analysis was applied in the evaluation of wine samples based on their geographical origin and ageing condition and the potential of excitation–emission matrices measurements by right angle geometry in combination with chemometrics was used for white wine classification, respectively [24,25].

In a previous work, we employed absorption spectroscopy for the analysis of Greek wines [26]. A total of 12 red and 10 white wines, all cultivated in Crete and matured for 3 months in various barrels, were analyzed by UV-Vis absorption spectroscopy. Using the spectroscopic data, the determination of the total phenolic index and the color analysis of red wines were accomplished. Moreover, multivariate data analysis and more specific Principal Components Analysis (PCA) was applied to the spectral data and a clear discrimination between wines has been obtained, according to the grape variety.

In the present work we further explore the applicability of UV-Vis absorption spectroscopy for the discrimination of Cretan monovarietal wines belonging to different grape varieties with a more comprehensive investigation, including a larger number of wine samples coming from three different vintage years and also from different producers. Herein, a total of 181 wine samples—66 from 2012, 88 from 2013 and 27 from 2018 vintage, respectively—were analyzed. The importance of the specific samples analysis lies on the fact that these wines have Protected Geographical Indication (PGI). Furthermore, the four monovarietal wines are made from indigenous varieties of Crete, two white (Dafni, Vilana) and two red (Mandilari, Kotsifali). For statistical analyses we initially used the unsupervised PCA method. The results obtained were quite satisfactory concerning the classification of samples. However, in the current study we employed the supervised Orthogonal Partial Least Squares—Discriminant Analysis (OPLS-DA) which shows improvements in the discrimination of wine samples compared to PCA method. Furthermore, from the OPLS-DA process, besides the score and loading plots, a confusion matrix is also derived, which quantitatively indicates the success rate of the classification achieved. In the current study we present the results obtained by application of OPLS-DA method on the spectral data of wine samples according to the grape variety, the time of maturation and the different container types.

## 2. Materials and Methods

### 2.1. Wine Samples

Samples consisted of four monovarietal wines, two white (Dafni, Vilana) and two red varieties (Mandilari, Kotsifali) all of which are cultivated in the region of Heraklion, Crete, Greece. The wine samples belonged to three vintages, two consecutive (2012, 2013) from the same wine producer and one recent (2018) from three other producers and from different cultivation fields. All the wine crops lie nearby Heraklion city. Different wooden barrels made of French oak, American oak, acacia and also stainless-steel containers with or without oak chips were used for the aging process. Samples from the different containers were analyzed at time intervals of 3 months. A total of 30 white wine samples and 36 red wine samples were collected for 2012 vintage. The number of white and red wine samples for 2013 vintage was 40 and 48, respectively, whereas for 2018 vintage the number of samples was 10 and 17 for white and red wines, respectively. Samples were stored at 16–18 °C and prior to the measurements were allowed to rise to room temperature. For the absorption measurements in the spectral region of ultraviolet (240–400 nm) the wine samples were diluted with ultrapure water. This dilution was made at 1:20 and 1:100 ratios for white and red wines, respectively. In the case of absorption measurements in the visible region (400–700 nm), wine samples were added to cuvettes without any dilution. 

### 2.2. Instrumentation

The absorption spectra of the wine samples were recorded on a Perkin Elmer Lambda 950 ultraviolet–visible (UV-Vis) spectrometer. The samples were placed in a quartz cuvette with a 10 mm pathlength. The absorbance was measured with a wavelength step of 2 nm in the spectral range of 240 to 700 nm. Ultrapure water was used for the reference scan. For the measurements in the UltraViolet (UV) spectral region, the dilution of the wine samples with ultrapure water was necessary in order for the absorbance intensity values to be within the 0.1–0.9 unit range and in accordance to the guidelines of wine analysis in the specific spectral region [27].

### 2.3. Machine Learning Procedures

The discrimination of the wine samples was performed by using multivariate statistical analysis techniques. The supervised statistical method of Orthogonal Partial Least Squares—Discriminant Analysis (OPLS-DA) was applied in the current study. OPLS-DA is a linear classification method that combines the properties of partial least squares regression with the discrimination power of a classification technique. The dimensionality of the initial independent variables (wavelengths) is reduced to a smaller number of latent (virtual) variables. Classification of samples is based on the highest probability of each sample to belong to a specific class [28,29]. 

The application of OPLS-DA results in score plots showing relations among the samples, thus leading to possible grouping of samples belonging to the same category.

Furthermore, employment of OPLS-DA produces loading plots that show relations between latent variables and the initial ones (wavelengths). Additionally, the confusion matrix derived from the process shows the rate of the successful classifications achieved by the method.

In many cases, it is necessary to pre-process spectral data, especially before multivariate statistical analysis [30]. After investigation and various tests among pre-processing methods, it was found that Singular Normal Variate transformation (SNV), mean center, first derivative and the Savitzky Golay smoothing method were appropriate for implementation; either each one alone, or in combination in this study.

The data analysis was performed by using Matlab R2013b (Mathworks, MA, USA) with the PLS Toolbox 8.1 (Eigenvector Research, Manson, WA, USA).

## 3. Results and Discussion

**Figure 1:**
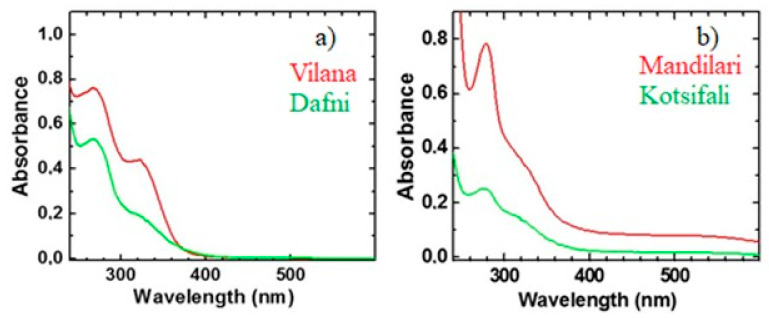
Typical Ultraviolet–Visible (UV-Vis) absorption spectra for (**a**) white wines (Dafni and Vilana) and (**b**) red wines (Kotsifali and Mandilari).

Typical UV-Vis spectra of four different wine samples are shown in Figure 1. From this figure it is obvious that for white wine varieties (Figure 1a), absorption bands occur in the ultraviolet region (268–280 nm and 320 nm), while for the red (Figure 1b), there is an extra absorption band in the visible region (500–524 nm). The chemical fingerprint of the specific Greek wine varieties has been discussed in a previous work [26]. Briefly, the absorption band at 280 nm is related to different types of phenolic compounds, while the absorption band at 320 nm is correlated to flavones and/or to nonflavonoid compounds. As far the red wines, the extra absorption band at about 520 nm corresponds to the presence of anthocyanin [31].

### 3.1. Discrimination of Grape Varieties

**Figure 2:**
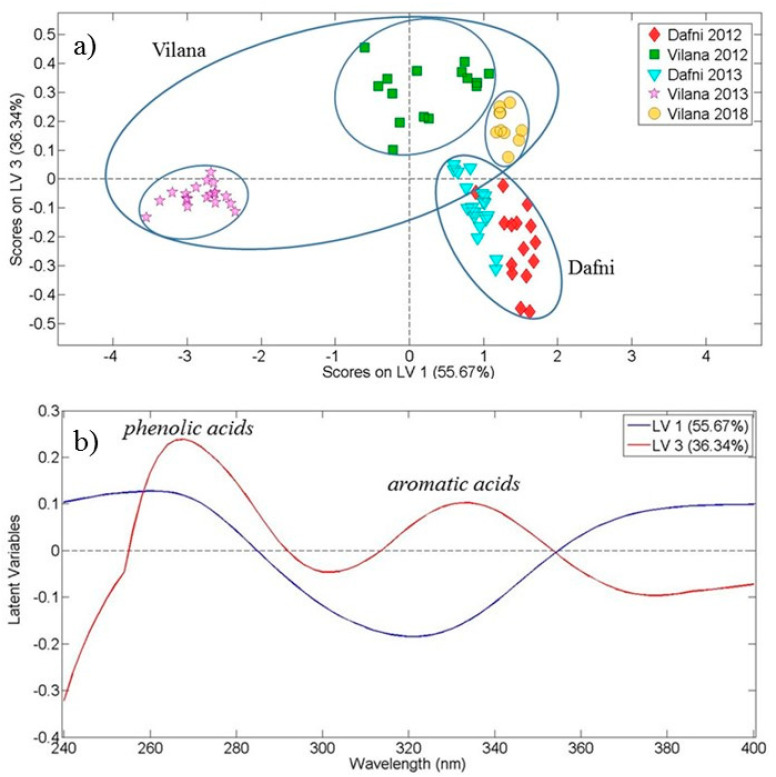
White wine Dafni and Vilana varieties discrimination. (**a**) Score plot of the first and the third latent variables resulting from the OPLS-DA application on ultraviolet spectral data of 80 white wine samples (2012, 2013 and 2018 vintages), and (**b**) The corresponding loading plot. Latent variable 1 is designated by the blue line and latent variable 3 by the red line.

For the discrimination of the wine samples based on the grape variety, a sophisticated supervised multivariate statistical method, such as OPLS-DA, was applied to the absorption spectral data from 240 to 400 nm, for white and red wines separately. The score plot of the first and third latent variable (Figure 2a) explained 92.01% of the total covariance in the case of 80 white wines of 2012, 2013 and 2018 vintages. A clear grouping among the two white grape varieties (Vilana, Dafni) was observed indicating the ability of absorption spectroscopy (UV spectral region) to discriminate wines produced from different grapes. It is worth noting that within the two main groups corresponding to the two white wine varieties, there exists subgroups which depict the different vintage years, as expected, but not with a systematic manner. During the current study we did not have access to samples of Dafni variety of the year 2018. The overlap between the two ellipses for Dafni and Vilana samples (Figure 2a) is due to the samples of Vilana 2018, even though, as extracted from the confusion table (Table 1) only two of the total of eighty samples were misclassified. Figure 2b shows the loading plot of the first and third latent variables as a function of wavelength in the ultraviolet region for the white varieties belonging to the 2012, 2013 and 2018 vintages.

The loading plot shows a negative correlation of the first latent variable in the wavelength region around 320 nm, while the third latent variable presents two positive correlations in the regions around 265 nm and 330 nm. It has been reported in the literature that the region around 260 nm is attributed to the absorption of non-flavonoid compounds (phenolic acids), while the region of 320 nm corresponds to flavonols and aromatic acids [24,32,33].

From the OPLS-DA model a confusion matrix/table can be extracted. This table describes the performance of a classification model on a group of data for which the true values are known. The results shown in Table 1 demonstrate a 97.50% model accuracy (successful classification) for the two investigated white grape varieties for three vintages (five different groups).

**Figure 3:**
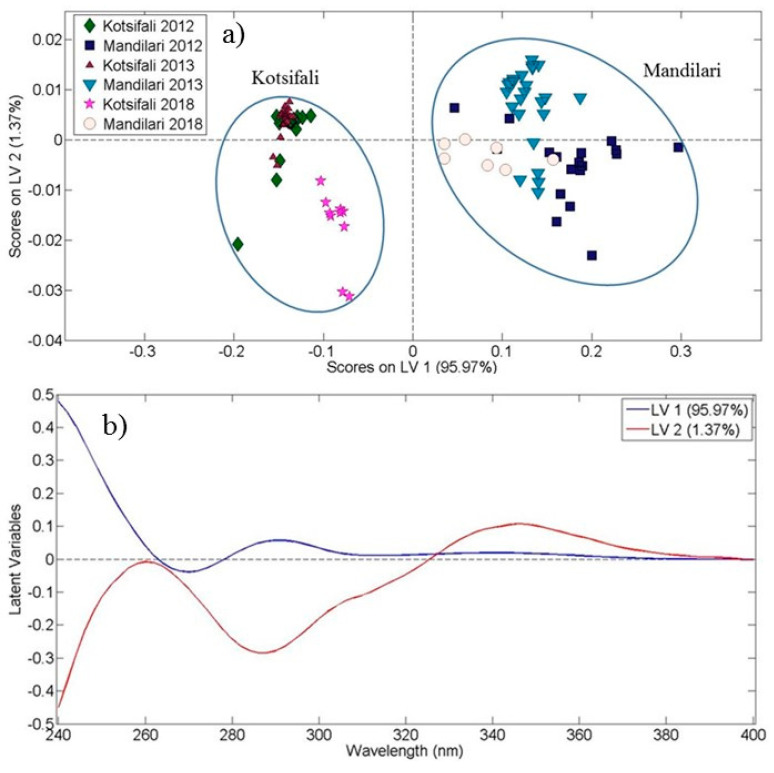
Red wine Kotsifali and Mandilari varieties discrimination. (**a**) Score plot of the first two latent variables resulting from the Orthogonal Partial Least Squares—Discriminant Analysis (OPLS-DA) application on ultraviolet spectral data of 101 red wine samples (2012, 2013 and 2018 vintages), and (**b**) The corresponding loading plot. Latent variable 1 is designated by the blue line and latent variable 2 by the red line.

Following the same procedure, we analyzed 101 red wine samples from the three vintages (2012, 2013 and 2018). The score plot of the first and second latent variable (Figure 3a) explained 97.34% of the total covariance. Using the statistical model (OPLS-DA) we have processed the absorption data from the ultraviolet region. A clear grouping among the two red grape varieties (Kotsifali, Mandilari) was observed. However, no clear subgrouping, indicating the different vintages inside the same red wine variety, was obvious, except from the Kotsifali samples of 2018, which group together and differentiate from the others.

The corresponding loading plot that resulted from OPLS-DA for red wines is presented in Figure 3b. The loading plot shows a positive correlation of the first latent variable in the wavelength region around 290 nm. The second latent variable presents one negative correlation at 290 nm and two positive ones at wavelengths around 260 nm and 345 nm. The latter may be attributed to the presence of flavonol groups (max absorption at 360 nm).

A confusion matrix/table is presented in the case of red wines. The results shown in Table 2 demonstrate a 93.07% model accuracy for the two studied red grape varieties for three vintages (six different groups).

### 3.2. Discrimination of Wines According to Aging Time 

**Figure 4:**
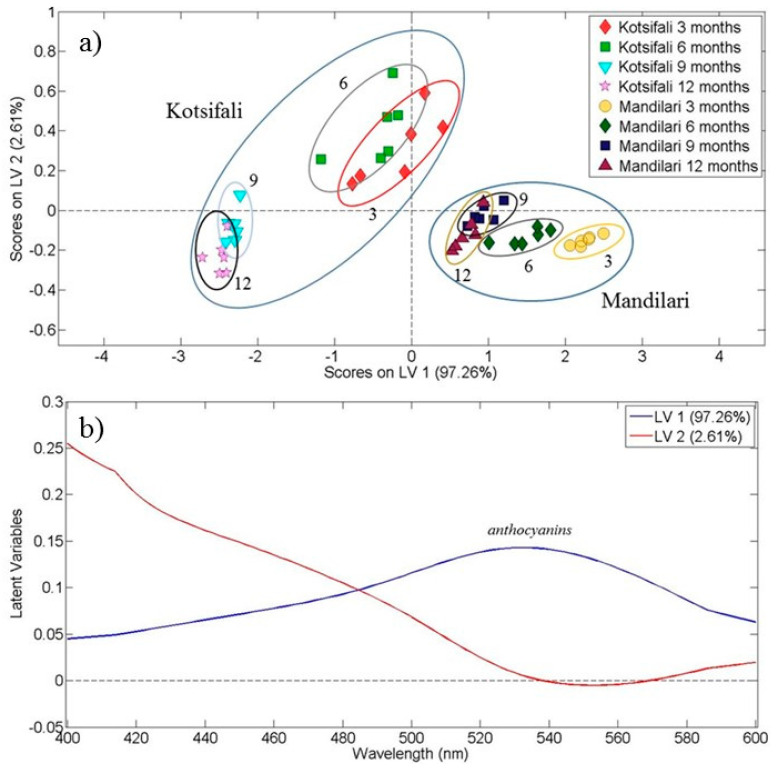
Red wine aging time (3, 6, 9 and 12 months) discrimination. (**a**) Score plot of the first and the second latent variables resulting from the OPLS-DA application on visible spectral data of 48 red wine Kotsifali and Mandilari samples (2013 vintage). The assignment of each category of wine samples is shown in the inset, and (**b**) Corresponding loading plot. Latent variable 1 is designated by the blue line and latent variable 2 by the red line.

Another parameter examined in this study was the discrimination of wine samples according to aging time in different type of containers. In Figure 4 we present the results of aging for the red varieties Kotsifali and Mandilari, since the aging of red wines is of main importance for the wine producers. In this case OPLS-DA was performed on the data from the visible region (400–700 nm) of absorption spectra for the red wines from the 2013 vintage. Figure 4a shows the score plot for the first two latent variables, which explained 99.71% of the total covariance. The samples of Kotsifali variety are situated on the left part of the diagram, with wines aged for 3 and 6 months creating a subgroup at the upper part, whereas samples aged for 9 and 12 months generate a second subgroup at the lower part, respectively. It is evident that there exists a systematic discrimination of aging time.

The samples belonging to Mandilari variety are positioned at the lower right part of the graph. Moreover, the samples of Mandilari variety are more confined than the Kotsifali ones. Τhis is possibly due to the initial high concentration of the total phenolic content for the specific red wine variety [26,34], which resulted in the lower contribution of aging time in the classification between Mandilari samples. This reflects the oenological behavior of these varieties since it is known that Mandilari has a higher phenolic content than Kotsifali [13,31]. For the case of Mandilari a more distinct discrimination among different aging times 3, 6, 9 and 12 months is observed. Contact time has a crucial role on wine chemical composition due to the different rate of extraction of many substances from the wood, e.g., aromatic compounds and ellagitannins [1]. It has recently been reported that the hydrolysable tannins released from oak chips reached a maximum value after 2 months [35].

The loading plot that resulted from OPLS-DA for red wines and at different contact times is presented in Figure 4b. The loading plot shows a positive correlation of the first latent variable in the wavelength region around 530 nm. The second latent variable presents a negative correlation at around 550 nm. It is well known that the absorption peak at around 520 nm in red wine is associated with anthocyanins [31].

Table 3 shows the OPLS-DA model accuracy in the case of red wine samples from 2013 vintage based on the contact time. The model accuracy is at the level of 72.92% (lower than the previous cases for varieties discrimination). Nevertheless, the predicted values of the statistical model are satisfactory and only few samples, which belong to continuous trimesters (i.e., 3 and 6 months for Kotsifali variety), were misclassified. The predicted values are better in the case of Mandilari samples.

### 3.3. Discrimination of Container Type

**Figure 5:**
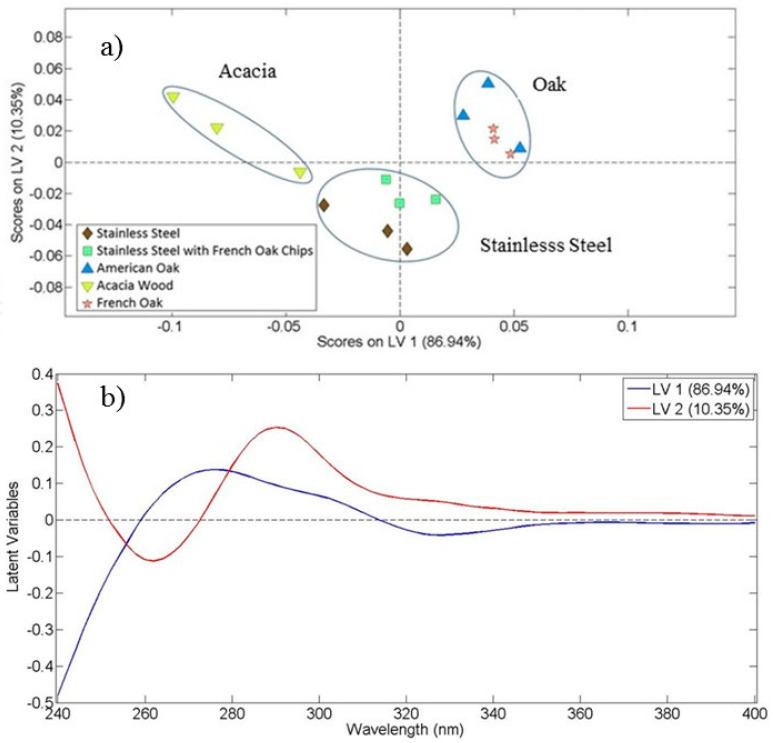
Maturation container discrimination. (**a**) Score plot of the first and the second latent variables resulting from the OPLS-DA application on ultraviolet spectral data of the white wine Vilana samples (2012 vintage). The assignment of each category of wine samples is shown in the inset, and (**b**) Corresponding loading plot. Latent variable 1 is designated by the blue line and latent variable 2 by the red line.

An important parameter affecting the wine quality is the type of the container where the wine is maturated. To this end, Figure 5a shows the OPLS-DA score plot resulting from the data in the ultraviolet region (240–400 nm) of absorption spectra for the white wine Vilana from the 2012 vintage, maturated in five different containers. At this point it should be mentioned that we performed the same analysis for all varieties of wine and we present herein indicative results for Vilana samples. A good clustering based on container (barrels and stainless steel) of maturation is shown, with the first and second latent variables explaining 97.38% of the total covariance in the 15 wine samples. From Figure 5a, three main groups emerge, which include the five different types of containers. The first group consists of wines aged in stainless steel containers (six samples), with and without the addition of French oak chips; the second group consists of wines aged in oak (French and American) barrels (six samples), and the third group includes wines aged in acacia barrels (three samples).

Moreover, Figure 5a shows that the first latent variable can separate samples aging in acacia barrels from samples aging in oak barrels, while the second latent variable can discriminate samples aging in stainless steel containers from those aging in barrels (oak, acacia).

Another general conclusion drawn from Figure 5a is that samples matured in acacia barrels are well separated from all the other samples, based on LV1. It has been reported in the literature that acacia wood contains low molecular weight phenolic compounds and a distinctive tannin profile and thus differs from the other types of wood [36].

The loading plot that resulted from OPLS-DA for white wine Vilana matured in different containers is presented in Figure 5b. The loading plot shows a positive correlation of the first latent variable in the wavelength region around 270 nm and a negative correlation at 325 nm. The second latent variable presents a negative correlation at 260 nm and a positive correlation at 290 nm.

Evaluation of the score and loading plots (Figure 5a,b) for the Vilana grape variety based on LV1 revealed that samples aged in acacia barrels present absorption at around 325 nm, while wine samples aged in stainless steel absorb mainly at around 260 nm, based on LV2.

Table 4 shows the OPLS-DA model accuracy in the case of Vilana white wine variety from 2012 vintage based on the container type discrimination. We have included samples aged in oak barrels (American and France) in one group and also samples contained in stainless steel (with and without oak chips) in another. The third group includes samples in the acacia barrel. A clear discrimination (100% accuracy for the three formed groups of wine) was accomplished.

## 4. Conclusions

This work establishes the capability of UV-Vis absorption spectroscopy for the classification of wines produced from different grape varieties. The discrimination ability of the method is further enhanced by the diversity of the samples used in this study, namely wine samples from three different vintage years, different wine producers and different fields of wine crops. A specific spectroscopic technique was used for the first time for the discrimination between wines aged in different containers and for different time periods. UV-Vis absorption spectroscopy is an alternative analytical tool in wine analysis and authentication as it is characterized by simplicity, rapidness, low cost and no sample pretreatment. The combination of spectroscopic results with the OPLS-DA statistical method resulted in a clear classification of samples with high values of model accuracy. A connection between families of chemical compounds and specific absorption ranges was considered based on the corresponding loading plots.

Further studies with a much larger number of samples from the same geographical region, also with protected geographical indication, will lead to the creation of a useful database containing the spectroscopic fingerprints of the local wines.

## Figures and Tables

**Table 1:** Classification of white grape varieties (2012, 2013 and 2018 vintages) with OPLS-DA.

	Dafni 2012	Vilana 2012	Dafni 2013	Vilana 2013	Vilana 2018
Predicted as Dafni 2012	**15**	0	1	0	0
Predicted as Vilana 2012	0	**15**	0	0	0
Predicted as Dafni 2013	0	0	**19**	0	1
Predicted as Vilana 2013	0	0	0	**20**	0
Predicted as Vilana 2018	0	0	0	0	**9**

**Table 2:** Classification of red grape varieties (2012, 2013 and 2018 vintages) with OPLS-DA.

	Kotsifali 2012	Mandilari 2012	Kotsifali 2013	Mandilari 2013	Kotsifali 2018	Mandilari 2018
Predicted as Kotsifali 2012	**16**	0	1	0	0	0
Predicted as Mandilari 2012	0	**18**	0	1	0	0
Predicted as Kotsifali 2013	2	0	**23**	0	0	0
Predicted as Mandilari 2013	0	0	0	**22**	0	1
Predicted as Kotsifali 2018	0	0	0	0	**10**	1
Predicted as Mandilari 2018	0	0	0	1	0	**5**

These results, for white and red wines, indicate the capability of absorption spectroscopy to discriminate Greek wines produced from different grapes.

**Table 3:** Classification of red wines (2013 vintage) as per aging time with OPLS-DA.

	Kotsifali 3 Months	Kotsifali 6 Months	Kotsifali 9 Months	Kotsifali 12 Months	Mandilari 3 Months	Mandilari 6 Months	Mandilari 9 Months	Mandilari 12 Months
Predicted as Kotsifali 3 months	**1**	3	0	0	0	0	0	0
Predicted as Kotsifali 6 months	5	**3**	0	0	0	0	0	0
Predicted as Kotsifali 9 months	0	0	**6**	1	0	0	0	0
Predicted as Kotsifali 12 months	0	0	0	**5**	0	0	0	0
Predicted as Mandilari 3 months	0	0	0	0	**5**	0	0	0
Predicted as Mandilari 6 months	0	0	0	0	0	**4**	1	0
Predicted as Mandilari 9 months	0	0	0	0	1	2	**5**	0
Predicted as Mandilari 12 months	0	0	0	0	0	0	0	**6**

**Table 4:** Classification of white variety (Vilana, 2012 vintage) concerning the container type with OPLS-DA.

	Stainless Steel	Acacia Wood	Oak Wood
Predicted as Stainless Steel	**6**	0	0
Predicted as Acacia Wood	0	**3**	0
Predicted as Oak Wood	0	0	**6**
